# Hydrogel-coated and active clearance chest drains in cardiac surgery: real-world results of a single-center study

**DOI:** 10.1186/s13019-024-02987-2

**Published:** 2024-08-23

**Authors:** Freya Sophie Jenkins, Mohammed Morjan, Jan-Philipp Minol, Nora Farkhondeh, Ismail Dalyanoglu, Esma Yilmaz, Moritz Benjamin Immohr, Bernhard Korbmacher, Udo Boeken, Artur Lichtenberg, Hannan Dalyanoglu

**Affiliations:** 1 Department of Cardiac Surgery, Medical Faculty, Heinrich Heine University, Dusseldorf, Germany; 2 Department of Cardiology, Sana Gerresheim Teaching Hospital, Dusseldorf, Germany; 3 Medical Faculty of the Semmelweiss University, Budapest, Hungary; 4 Department of Cardiac Surgery, University Hospital Dusseldorf, Moorenstr. 5, 40225 Dusseldorf, Germany

**Keywords:** Chest drains, Pleural effusion, Pneumothorax, Pericardial effusion, Postoperative arrhythmias

## Abstract

**Purpose:**

Cardiac surgery patients require chest drains for postoperative fluid drainage. Innovations in this field include chemical drain coating and manual clot extraction systems, aiming to provide reduced clotting and improved patient comfort. This study compares outcomes using hydrogel-coated, active clearance and conventional chest drains.

**Methods:**

Patients with cardiac surgery at our institution from January 2023 to September 2023 were included. Drain allocation was based on surgeon’s choice, with either a combination of hydrogel-coated and conventional, active clearance and conventional, or conventional drains alone. Drain data and clinical outcomes were recorded prospectively.

**Results:**

One hundred seventy-eight patients (62.9 ± 11.7 years, 67.4% male) received a total of 512 chest drains intraoperatively. Hydrogel-coated and active clearance drains showed higher drainage volumes than conventional drains (*p* < 0.001, respectively). Patency was lowest in conventional drains (36.7% vs. 98.8% for hydrogel-coated, *p* < 0.001, and vs. 96.6% for active clearance drains, *p* < 0.001). Conventional drains showed 5.9 times the odds (95% CI 2.0–25.2) of large pleural effusions compared to hydrogel-coated and 12.0 times the odds (95% CI 1.9–504.1) compared to active clearance drains. Patients with hydrogel-coated drains had the shortest length of stay (*p* < 0.001).

**Conclusion:**

Hydrogel-coated and active clearance drains show improved outcomes compared to conventional drains in cardiac surgery.

## Background

Chest drains are inserted intraoperatively in all cardiac surgery patients to allow for postoperative drainage of blood and serous fluids. Depending on the cardiosurgical procedure, drains may be positioned in the pericardial space, in the pleural space and/or in the substernal space. Maintaining drain patency is considered paramount to prevent retention of blood and fluids, and to reduce the requirement for subsequent additional intervention [1, 2]. However, chest drains are prone to clogging with blockage often due to intrathoracic blood clots that are only visible once the drain has been removed [3]. Strategies to combat blockage of conventional chest drains include “milking” and “stripping”, in which a healthcare professional attempts to free the drain of clots and debris by creating a negative pressure gradient between the intrathoracic part of the drain in the patient and the outside part, without breaking the sterile environment. A further method is to disconnect the drain and advance a smaller catheter with active suction to pull out clots and debris. These strategies can cause tissue damage, infections from breaking the sterile environment and pneumothorax, and are not recommended in the guidelines on postoperative care of cardiac surgery patients [1, 4, 5].

Indwelling chest drains are associated with significant patient discomfort and can delay postoperative mobilization [6]. In addition, initiation of oral anticoagulants may be deferred until all large catheters, including chest drains, are removed, although there is no consensus on the optimal timing of chest drain removal [7]. In clinical practice, decision to remove a drain is often based on a combination of time since surgery, drainage volume and x-ray imaging, with pericardial and substernal chest drains generally removed before pleural ones. X-ray imaging plays a particular role in deciding on the removal of pleural drains, as a clogged drain may show no drainage volume although a relevant pleural effusion is visible radiologically. Although x-ray is the standard diagnostic technique for assessment of pleural effusion, interpretation in postoperative cardiac surgery patients can be more difficult [8]. Studies investigating the timing of chest drain removal have shown higher rates of pericardial and pleural effusions when chest drains are removed earlier [9, 10]. In contrast, longer chest drain duration has been associated with longer time on the intensive care unit (ICU) and longer hospital stay [11].

Innovative technologies in the field of chest drains include coating the drain with a hydrogel polymer to prevent clot adhesion in the drain and in-built active clearance systems that allow removal of clots by advancing an intraluminal catheter without breaking the sterile environment. Several studies have found benefits of using active clearance chest drains, including reduced rates of surgical re-exploration, reduced intrathoracic blood retention and lower rates of postoperative atrial fibrillation when compared to conventional chest drains [12–16]. In contrast, other studies have found no benefit of active clearance system drains, including no reduction in re-exporation, intervention for pneumothorax and intervention for pleural effusion [17, 18]. The literature to date lacks investigation of hydrogel-coated chest drains and their comparison with both conventional and active clearance system chest drains.

Our study aimed to examine hydrogel-coated, active clearance system and conventional chest drains in a cohort of cardiac surgery patients at a single institution.

## Methods

### Study patients and chest drains

Patients undergoing any type of major cardiac surgery from January 2023 to September 2023 at our institution were included in the analysis. Patients received the number and positioning of intraoperative chest drains according to institutional practice for the given surgical procedure. Based on the surgeon’s preference, patients received one of three drain combinations: a combination of hydrogel-coated drains (ClotStop^®^, Axiom Medical Inc., Torrance, CA, USA) and conventional drains, a combination of active clearance system drains (PleuraFlow^®^ ACT^®^, ClearFlow Inc., Irvine, CA, USA) and conventional drains, or conventional drains alone. For hydrogel-coated drains, 28 French drains were inserted pericardially and 32 French drains in the pleural and substernal space. Due to availability, all active clearance system drains used in the study were 32 French, independent of anatomical location. The conventional drains used were in line with institutional practice and included 27 French silicone drains in the substernal position (Dahlhausen Medizintechnik, Cologne, Germany), 30 French drains in the pericardial position (Andocor, Hoogstraten, Belgium) and 32 French drains (Andocor, Hoogstraten, Belgium) in the pleural position, with choice of size based on surgeon’s preference.

### Study design

The study was of non-randomized prospective design. Patients were followed during their hospital stay and relevant data on chest drains and clinical outcomes were recorded in a predefined central database.

### Ethics

The study followed the principles of the Declaration of Helsinki and was approved by the Ethics Committee of Heinrich-Heine University, Dusseldorf (study number: 2023–2629).

### Chest drain assessment and removal

Chest drain management was performed according to institutional practice for conventional and hydrogel-coated drains. For active system clearance drains, removal of clots with the clearance system was performed at regular intervals. All drains were assessed for drainage volume, respiratory tidaling and manipulation at ICU arrival, two hours after ICU arrival, twelve hours after ICU arrival and at removal. The decision to remove the drain was based on institutional practice for all drains. Freedom from clots was evaluated visually after removal.

### Clinical outcomes

The main clinical outcomes of the study were the incidence of pleural effusions, pericardial effusions, bronchopulmonary infections, postoperative arrhythmias from admission to ICU until discharge from hospital, and length of stay. The assessment of effusions was performed on a by-drain basis. Hence, for an effusion to be counted as relevant to the drain type, it had to occur in the same anatomical location as the drain in question. For example, a patient after coronary artery bypass grafting with one left-sided chest drain and one substernal drain was considered to have a study-related pleural effusion if it occurred on the ipsilateral, i.e. left, side. Effusions were divided based on their management into conservatively managed and those requiring intervention.

### Data analysis

Descriptive statistics for normally distributed quantitative variables are summarized as means with standard deviation. For skewed data, the median is shown. Categorical data is presented with proportions. Odds ratios and their 95% confidence intervals were calculated using contingency tables and Fisher’s exact test. The Haldane-Anscombe correction was used for odds ratio calculations with zero values in contingency tables. Statistical significance was established as *p* < 0.05 with the Bonferroni correction applied for multiple testing. Analysis and evaluation of data were carried out using the statistical software R, version 4.3.2 (R Foundation for Statistical Computing, Vienna, Austria).

## Results

### Patients and chest drains

A total of 512 chest drains in 178 patients (mean age 62.9 ± 11.7, 67.4% male) were included in the analysis. Of the 178 patients, 54 received at least one hydrogel-coated drain, 58 at least one active clearance system drain and 66 patients received conventional drains only. No patient received a combination of hydrogel-coated and active clearance chest drains. The patient groups differed for rates of chronic obstructive pulmonary disease, which was significantly more common in patients with conventional drains only (21.2% vs. 3.7% for patients with at least one hydrogel-coated drain, *p* = 0.010). Impaired left ventricular ejection fraction (LVEF) was more common in patients in the active clearance chest drain group and these patients also had higher rates of preoperative antiplatelet and anticoagulant therapy than those in the hydrogel-coated and conventional chest drain group. (Table 1). The most common surgery overall involved the thoracic aorta with or without the aortic valve, followed by surgery of the coronary arteries and implantation of a left ventricular assist device (LVAD). Mean number of drains per patient was 2.9 ± 0.8. Type of surgery differed between the patient groups, with those patients with at least one active clearance system drain having a higher proportion of aortic valve and aortic surgery, and LVAD implantation. Of the 512 drains, 95 (18.6%) were coated with a hydrogel polymer, 87 (17.0%) included an active clearance system and 330 drains (64.5%) were conventional. (Table 2).

**Table 1:** Patient characteristics

Variable	1 or more HC	1 or more ACS	CD only	*p*-value		
	(*n* = 54)	(*n* = 58)	(*n* = 66)	**HC vs.** **CD**	**ACS vs. CD**	**HC vs. ACS**
Age, mean in years	63.2	61.4	63.9	0.73	0.26	0.41
Male sex (%)	70.4	75.9	57.6	0.21	0.05	0.66
BMI, mean	26.2	26.7	26.5	0.54	0.85	0.69
Ever-smoker (%)	18.5	20.7	27.3	0.36	0.52	0.96
COPD (%)	3.7	10.3	21.2	**0.01**	0.16	0.32
Creatinine, mean in mg/dL	1.10	1.19	1.27	0.39	0.55	0.62
Previous heart surgery (%)	7.4	13.8	10.6	0.58	1.00	0.61
Preoperative LVEF less than 55% (%)	35.2	62.1	53.0	0.08	**< 0.01**	**< 0.01**
Hemostasis at admission (%)						
Aspirin (%)	11.1	37.9	19.7	0.30	**< 0.01**	**< 0.01**
Vitamin K antagonist (%)	1.8	27.6	3.0	0.60	**< 0.001**	**< 0.001**
International normalized ratio, mean	1.2	1.6	1.3	0.52	**0.02**	**< 0.01**
Activated thromboplastin time (sec), mean	29.5	29.3	29.1	0.94	0.87	0.86
Type of surgery (%)				0.49	**< 0.01**	**< 0.01**
SAVR and aortic surgery	27.8	60.3	21.2			
CABG	44.6	10.3	42.4			
LVAD implantation	1.9	29.3	4.5			
Mitral valve replacement	18.5	0.0	13.6			
Heart transplantation	5.6	0.0	15.2			
Other	1.9	0.0	3.0			
Surgery duration (mins), mean	223	282	254	0.09	0.27	**0.01**
Number of drains, mean	2.7	2.8	3.1	**< 0.01**	0.07	0.24

BMI = Body mass index. CABG = Coronary artery bypass grafting. LVAD = Left ventricular assist device. LVEF = Left ventricular ejection fraction. SAVR = Surgical aortic valve replacement. SD = Standard deviation. Significance is indicated by bold values

**Table 2:** Anatomical location of chest drains

Location	HC	ACS			CD	
Pleural	49	30			144	
Pericardial	4	13			119	
Substernal	42	44			67	
Total	95	87			330	

ACS = Active clearance system drains. CD = Conventional drains. HC = Hydrogel-coated drains

### Drainage volume

**Fig. 1:**
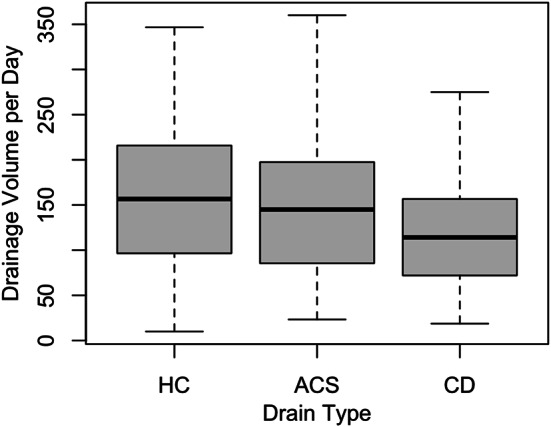
Comparison of drainage volume per day by drain type. ACS = Active clearance system drains. CD = Conventional drains. HC = Hydrogel-coated drains

Conventional drains showed the lowest drainage volume independent of anatomical drain location. (Table 3). Specifically, twelve hours after arrival on the ICU, conventional drains in the pleural position had drained 120 mL compared to 200 mL for hydrogel-coated drains (*p* < 0.001) and 190 mL for active clearance system drains (*p* < 0.001). For pericardial drains, the respective results showed 130 mL for conventional drains compared to 330 mL for hydrogel-coated drains (*p* = 0.001) and 210 mL for active clearance system drains (*p* < 0.001). For substernal drains, drainage volumes twelve hours after arrival on the ICU were 100 mL for conventional drains compared to 145 mL for hydrogel-coated drains (*p* < 0.001) and 150 mL for active clearance system drains (*p* < 0.001). There was no difference in drainage volume after twelve hours between hydrogel-coated and active clearance system drains in the pleural (*p* = 0.86), pericardial (*p* = 0.17) or substernal (*p* = 0.96) location. Drainage volume per day was highest in the hydrogel-coated drains (mean 180.8 mL ± 148.7; median 156.7 mL), followed by the active clearance system drains (mean 162.2 mL ± 121.1, median 145.0 mL) and the conventional drains (mean 129.5 mL ± 104.1, median 114.2 mL) (*p* < 0.001 for hydrogel-coated vs. conventional drains, and *p* = 0.003 for active clearance system vs. conventional drains). (Fig. 1). Conventional drains consistently showed the lowest drainage volume per day independent of anatomical drain location. (Table 3).

**Table 3:** Drainage volume, time to removal, manipulation and patency

Variable	HC	ACS	CD	*p*-value		
				HC vs. CD	ACS vs. CD	HC vs. ACS
Drainage in 12 h in mL, median						
Pleural	200	190	120	** <0.01**	**<0.01**	0.86
Pericardial	330	210	130	** <0.01**	**<0.01**	0.17
Substernal	145	150	100	**<0.01**	**<0.01**	0.96
Drainage volume per day, median						
Pleural	157	143	125	0.17	0.73	0.36
Pericardial	170	187	110	**0.03**	**0.02**	0.78
Substernal	150	143	80	**< 0.001**	**< 0.001**	0.50
Time to removal in days, mean						
Pleural	4.0	4.9	5.8	** <0.01**	0.18	**0.01**
Pericardial	4.8	4.2	2.7	0.47	**0.01**	0.95
Substernal	2.3	3.0	2.6	0.08	0.09	**<0.01**
Manipulation at any time (%)						
Pleural	10.2	96.7	47.9	**<0.01**	**<0.01**	**<0.01**
Pericardial	0.0	100.0	53.8	0.11	**<0.01**	**<0.01**
Substernal	4.8	100.0	38.8	**<0.01**	**<0.01**	**<0.01**
Tidaling at removal (%)						
Pleural	100.0	90.0	41.0	**<0.01**	**<0.01**	0.10
Pericardial	50.0	100.0	26.9	0.65	**<0.01**	0.07
Substernal	97.6	100.0	44.8	**<0.01**	**<0.01**	0.98
Free of clots at removal (%)						
Pleural	93.9	93.3	53.5	**<0.01**	**<0.01**	1.00
Pericardial	50.0	0.0	50.4	1.00	**<0.01**	0.07
Substernal	83.3	2.3	40.3	**<0.01**	**<0.01**	**<0.01**

ACS = Active clearance system drains. CD = Conventional drains. HC = Hydrogel-coated drains. Significance is indicated by bold values

### Time to drain removal

Overall time to drain removal varied based on anatomical location of the drains. Drains in the substernal location were removed earliest at 2.6 ± 1.4 days, followed by drains in the pericardial location at 2.9 ± 1.6 days, and pleural drains at 5.3 ± 2.8 days. Hydrogel-coated clots were removed after the fewest number of days in the pleural location compared to the active clearance system and conventional drains. (Table 3).

### Drain manipulation

As per their intrinsic design, almost all active clearance drains underwent some form of manipulation for clot removal before they were removed from the patient. Compared to hydrogel-coated drains, conventional drains were manipulated significantly more often independent of anatomical location. (Table 3).

### Drain patency and visible clots

Patency as measured by respiratory tidaling at removal was higher in drains with hydrogel coating than in conventional drains (98.8% vs. 36.7%, *p* < 0.001) and was also higher in drains with active clearance systems compared to conventional drains (96.6% vs. 36.7%, *p* < 0.001). (Table 3). The difference became marked twelve hours after arrival on the ICU, when 26.7% of conventional drains no longer showed respiratory tidaling (compared to 2.1% with hydrogel coating, *p* < 0.001 and 3.4% for active clearance systems, *p* < 0.001). Hydrogel-coated drains in the pleural position showed 100% respiratory tidaling at removal (compared to 41.0% for conventional drains, *p* < 0.001 and 90.0% for active clearance drains, *p* = 0.100). There was no difference for respiratory tidaling between hydrogel-coated and active clearance drains (*p* = 1.000). On removal, hydrogel-coated drains were most frequently free of clots, at a rate of 87.4%, compared to 49.7% for conventional drains (*p* < 0.001), and 33.3% for active clearance drains (*p* < 0.001). Manipulation at any time was positively associated with respiratory tidaling at removal in conventional drains (*p* < 0.001) but not in hydrogel-coated (*p* = 0.531) or active clearance systems drains (*p* = 1.000). In contrast, freedom of clots at removal was not associated with previous manipulation in any of the drain types.

### Clinical outcomes by chest drain

**Fig. 2:**
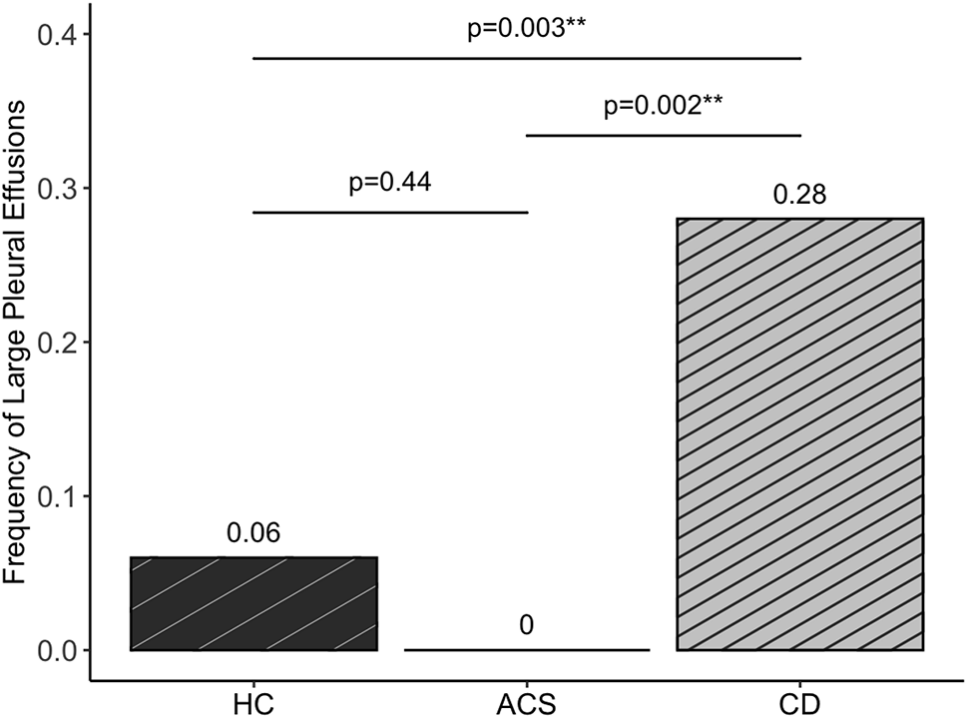
Large pleural effusions by drain type before discharge. ACS = Active clearance system drains. CD = Conventional drains. HC = Hydrogel-coated drains

Occurrence of conservatively managed ipsilateral pleural effusions before discharge was comparable for all drains. (Table 4). Large ipsilateral pleural effusions requiring additional drainage were less common in hydrogel-coated compared to conventional (6.1% vs. 27.8%, *p* = 0.003) and in active clearance drains compared to conventional drains (0.0% vs. 27.8%, *p* = 0.002). (Fig. 2). Conventional drains showed 5.90 times the odds (95% CI 2.01–25.24) of large pleural effusion compared to hydrogel-coated drains and 12.01 times the odds (95% CI 1.87–504.07) compared to active clearance system drains. There was no difference in the rates of large pleural effusions between hydrogel-coated and active clearance drains (*p* = 0.44). For conventional drains, manipulation at any time was protective against large pleural effusions (OR 0.35, 95% CI 0.16–0.77). Across all drains, freedom from clots at removal was protective against pleural effusions (OR 0.47, 95% CI 0.24–0.93). Chronic obstructive pulmonary disease preoperatively was not associated with postoperative conservative pleural effusion (*p* = 0.427) or those needing intervention (*p* = 0.155). Type of surgery was also not associated with occurrence of postoperative pleural effusions (*p* = 0.920). Neither was preoperative intake of aspirin (*p* = 0.84) or vitamin K antagonists (*p* = 0.07). Patients with higher international normalized ratio or higher activated partial thromboplastin time at admission did not show pleural effusions more frequently (*p* = 0.73 and *p* = 0.31, respectively). Out of the total 178 patients in our study, 7 (3.9%) experienced an overt postoperative coagulation disorder. The rate of pleural effusion requiring intervention did not differ between these patients and those without a coagulation disorder (*p* = 0.09). Preoperative impaired left ventricular ejection fraction was not associated with higher rates of postoperative pleural effusions (*p* = 0.08 for conservatively managed and *p* = 1.00 for those requiring intervention) and neither was the need for mechanical life support (*p* = 1.00 and *p* = 0.56, respectively). The rates of pneumothorax, both with conservative and interventional management, were comparable across all drains. Conventional drains showed a tendency towards more bronchopulmonary infections than hydrogel-coated and active clearance system drains. Bronchopulmonary infections were not associated with rates of manipulation or longer time to removal. (Table 4).

**Table 4:** Clinical outcomes before discharge

Variable	HC	ACS	CD	*p*-value		
				HC vs. CD	ACS vs. CD	HC vs. ACS
Pleural (%)						
Pleural effusion, conservative	30.6	30.0	43.1	0.17	0.26	1.00
Pleural effusion, intervention	6.1	0.0	27.8	**<0.01**	**<0.01**	0.44
Pneumothorax, conservative	0.0	6.7	4.9	0.26	1.00	0.27
Pneumothorax, intervention	0.0	0.0	2.8	0.55	0.80	1.00
Bronchopulmonary infection	6.1	6.7	13.2	0.28	0.49	1.00
Pericardial (%)						
Pericardial effusion, conservative	25.0	30.8	19.3	1.00	0.54	1.00
Pericardial effusion, intervention	0.0	0.0	4.2	1.00	1.00	1.00
Postoperative arrhythmia	0.0	38.5	30.3	0.45	0.77	0.40

ACS = Active clearance system drains. CD = Conventional drains. HC = Hydrogel-coated drains. Significance is indicated by bold values

The rate of conservatively managed pericardial effusions was 23.6% in our cohort and pericardial effusions requiring intervention occurred in 2.8% of patients. There was no difference in rates of small pericardial effusions with the different drains. Patients with hydrogel-coated and active clearance system drains in the pericardial location showed no pericardial effusions requiring intervention, compared to 4.2% of patients with conventional pericardial drains, although this result did not achieve statistical significance (*p* = 1.000). Occurrence of pericardial effusions was not associated with type of surgery (*p* = 0.67), but patients with a heart transplant were more likely than other patients to require an intervention for their pericardial effusion (*p* = 0.01). None of the patients with a postoperative coagulation disorder experienced a pericardial effusion requiring intervention, and need for postoperative mechanical life support was also not associated with pericardial effusion (*p* = 0.60 for conservatively managed effusions and *p* = 0.55 for those requiring intervention). Postoperative arrhythmias occurred in none of the patients with hydrogel-coated drains, compared to 38.5% with active system clearance drains and 30.3% with conventional drains. Similarly, this result did not achieve statistical significance. Postoperative arrhythmias did not show an association with pericardial effusions (*p* = 0.507).

### Length of stay

Mean length of stay for all patients was 23.1 ± 18.0 days. Patients with at least one hydrogel-coated drain had the shortest length of stay at 17.0 days, followed by 21.5 days for patients with at least one active clearance system drain (*p* < 0.001 compared to hydrogel-coated drains) and 29.4 days for patients with conventional drains only (*p* < 0.001 compared to hydrogel-coated drains and *p* = 0.011 compared to active clearance system drains). Occurrence of postoperative pleural effusion was associated with longer hospital stay, regardless of whether the effusion could be managed medically (*p* < 0.001) or required surgical intervention (*p* < 0.001).

## Discussion

Our study’s main findings are: (1) chest drains with hydrogel coating or an active clearance system show a reduced incidence of large pleural effusions compared to conventional chest drains; (2) hydrogel-coated drains in the pleural position were removed earlier than active clearance system and conventional drains; (3) there was no difference in the incidence of pleural effusions between hydrogel-coated drains and active clearance system drains and (4) hydrogel-coated and active clearance system drains drained more fluid per day than conventional drains. As a secondary finding, drain manipulation was associated with lower incidence of large pleural effusions in our study, as was freedom from clots at the time of removal.

The findings of our study suggest that innovative chest drains aimed at reducing drain blockage are associated with improved drainage and better clinical outcomes than conventional drains. Studies on the active clearance system drains have not consistently shown their benefit over conventional drains, with some authors questioning their value for clinical use after cardiac surgery and additional concerns related to higher cost of the drains and increased nursing time for active clearance [17, 18]. Our study did not find benefits of active clearance system drains for pericardial and substernal drainage, and although not statistically significant, active clearance system drains showed the highest rate of pericardial effusions and postoperative arrhythmias. However, our study showed a clear benefit of active clearance system drains in preventing large pleural effusions and a shorter time to removal compared to conventional drains. In our study, patients with at least one hydrogel-coated drain and patients with conventional drains underwent a significantly higher proportion of heart transplant and coronary artery bypass grafting than patients with at least one active clearance system drain, who showed a larger proportion of LVAD implantation. Coronary artery bypass grafting and heart transplant have been shown to be associated with higher occurrence of postoperative effusion [19]. However, in our study, patients with at least one active clearance chest drain were more likely to have preoperatively impaired left ventricular ejection fraction, which can also be considered a risk factor for development of postoperative effusion.

From an economic standpoint, the innovative drain types are significantly more expensive to purchase, with the active clearance system and hydrogel-coated drains costing between two to three hundred euros in Germany, and the conventional drains generally costing less than ten euros. As mentioned above, this may represent a significant hurdle for hospitals to adopt innovative drain types as their standard of care. However, a more holistic view of the costs that includes the indirect costs of each drain type is warranted. In this sense, achieving a reduction in length of stay as seen in patients with hydrogel-coated and active clearance system in our study is likely to offset any costs associated with the drain purchase. Similarly, time saved from drain manipulation is likely to lead to an overall cost saving. Further studies focusing on the health economic aspects of the different drain types would be helpful to this end.

Given the paucity of literature on hydrogel-coated drains in particular, our study provides new information on the clinical benefits of this type of drain. Based on our results, pleurally positioned hydrogel-coated drains show a significantly shorter time to removal compared to conventional and active system clearance drains. The hydrogel-coated drains also required significantly less manipulation overall without compromising on patency and clinical outcomes.

### Limitations

Our study has some limitations that should be mentioned. Associations between independent variables and dependent variables cannot be shown to be causal in nature. Allocation of drain types to patients was not based on randomization but on surgeon’s choice and availability of the drain at the time of the respective surgery. We consider it unlikely for this have significantly influenced our findings, given that surgeons used the innovative drains in a similarly proportionate way (average proportion of hydrogel-coated drains = 34.4%, and average proportion of active clearance drains = 33.0%). Due to the nature of our study, patients were unmatched and showed different in rates of impaired left ventricular ejection fraction preoperatively, COPD, antiplatelet and anticoagulation at admission, and the type of surgery performed. Although we cannot exclude a certain bias owing to this, the characteristics differing between the groups were not independently associated with the outcomes studied. In addition, due to the low number of certain events in our cohort, for example pneumothoraxes and pericardial effusions, identification of statistically significant differences was hard to achieve. Our study included only a small number of hydrogel-coated and active clearance system drains in the pericardial position, hindering robust conclusions about the effect of drain type in this anatomical location.

## Conclusions

Both hydrogel-coated and active clearance system drains appear to offer significant benefits compared to conventional drains in cardiac surgery. Overall, hydrogel-coated drains may be associated with more pronounced clinical benefits and lower resource intensity.

## Data Availability

No datasets were generated or analysed during the current study.

## Abbreviations

ACS: Active clearance system
BMI: Body mass index
CABG: Coronary artery bypass grafting
CD: Conventional drains
CI: Confidence interval
HC: Hydrogel-coated
ICU: Intensive Care Unit
LVAD: Left ventricular assist device
LVEF: Left ventricular ejection fraction
OR: Odds ratio
SAVR: Surgical aortic valve replacement
SD: Standard deviation
